# Evaluation of COVID-19 vaccine implementation in a large safety net health system

**DOI:** 10.3389/frhs.2023.1152523

**Published:** 2023-06-05

**Authors:** Jennifer C. Chen, Griselda Gutierrez, Rachel Kamran, Jill Terry, Armenui Telliyan, Camilo Zaks, Savanna L. Carson, Arleen Brown, Karen Kim

**Affiliations:** ^1^ Ambulatory Care Network, Los Angeles County Department of Health Services, Los Angeles, CA, United States; ^2^ Department of Emergency Medicine, David Geffen School of Medicine at UCLA, Los Angeles, CA, United States; ^3^ Harbor-UCLA Medical Center, Los Angeles County Department of Health Services, Los Angeles, CA, United States; ^4^ Department of Obstetrics and Gynecology, David Geffen School of Medicine at UCLA, Los Angeles, CA, United States; ^5^ Fielding School of Public Health Graduate Program, University of California, Los Angeles, CA, United States; ^6^ Pharmacy Affairs, Los Angeles County Department of Health Services, Los Angeles, CA, United States; ^7^ Adjunct Faculty, USC School of Pharmacy, Los Angeles, CA, United States; ^8^ Adjunct Nursing Faculty, Glendale Community College, Glendale, CA, United States; ^9^ Adjunct Nursing Faculty, West Coast University, Los Angeles, CA, United States; ^10^ Division of Street Medicine, Keck School of Medicine of USC, Los Angeles, CA, United States; ^11^ Division of General Internal Medicine & Health Services Research, David Geffen School of Medicine at UCLA, Los Angeles, CA, United States; ^12^ Population Health Management, Los Angeles County Department of Health Services, Los Angeles, CA, United States; ^13^ Department of Medicine, David Geffen School of Medicine at UCLA, Los Angeles, CA, United States

**Keywords:** COVID-19, vaccine, vaccine distribution, implementation, leadership, communication, integrated delivery of health care, equity

## Abstract

**Objectives:**

To evaluate rapid COVID-19 vaccine clinic implementation from January-April 2021 in the Los Angeles County Department of Health Services (LACDHS), the second-largest US safety net health system. During initial vaccine clinic implementation, LACDHS vaccinated 59,898 outpatients, 69% of whom were Latinx (exceeding the LA County Latinx population of 46%). LACDHS is a unique safety net setting to evaluate rapid vaccine implementation due to system size, geographic breadth, language/racial/ethnic diversity, limited health staffing resources, and socioeconomic complexity of patients.

**Methods:**

Implementation factors were assessed through semi-structured interviews of staff from all twelve LACDHS vaccine clinics from August-November 2021 using the Consolidated Framework for Implementation Research (CFIR) and themes analyzed using rapid qualitative analysis.

**Results:**

Of 40 potential participants, 25 health professionals completed an interview (27% clinical providers/medical directors, 23% pharmacist, 15% nursing staff, and 35% other). Qualitative analysis of participant interviews yielded ten narrative themes. Implementation facilitators included bidirectional communication between system leadership and clinics, multidisciplinary leadership and operations teams, expanded use of standing orders, teamwork culture, use of active and passive communication structures, and development of patient-centered engagement strategies. Barriers to implementation included vaccine scarcity, underestimation of resources needed for patient outreach, and numerous process challenges encountered.

**Conclusion:**

Previous studies focused on robust advance planning as a facilitator and understaffing and high staff turnover as barriers to implementation in safety net health systems. This study found facilitators that can mitigate lack of advance planning and staffing challenges present during public health emergencies such as the COVID-19 pandemic. The ten identified themes may inform future implementations in safety net health systems.

## Introduction

When COVID-19 vaccines attained U.S. Food and Drug Administration Emergency Use Authorization (EUA) and were made available to U.S. health systems in December 2020, safety net health systems were challenged to implement widespread vaccination in resource-limited environments during a time of peak COVID-19 transmission (1, 2). Vaccination implementation entailed understanding the evolving regulations of vaccine eligibility and availability, then distributing vaccine accordingly to vulnerable communities experiencing significant racial and economic inequities due to the COVID-19 pandemic. Implementation of safety net initiatives has been associated with challenges including limited staffing, lack of organizational financial investment, and the need to address patients' biopsychosocial complexities. Facilitators to implementation have included advance planning, redundancy in communication, knowledge of patient needs, desire to perform well, personnel commitment to reducing health inequities, and multidisciplinary teams to drive implementation (3–6). More research is needed to understand the role of implementation factors in the safety net, particularly for primary care-led vaccine distribution approaches (7). To date, there are few published qualitative studies of COVID-19 vaccine delivery in safety net health systems (8, 9), and none in a large safety net health system with coordination across many sites.

**Figure 1:**
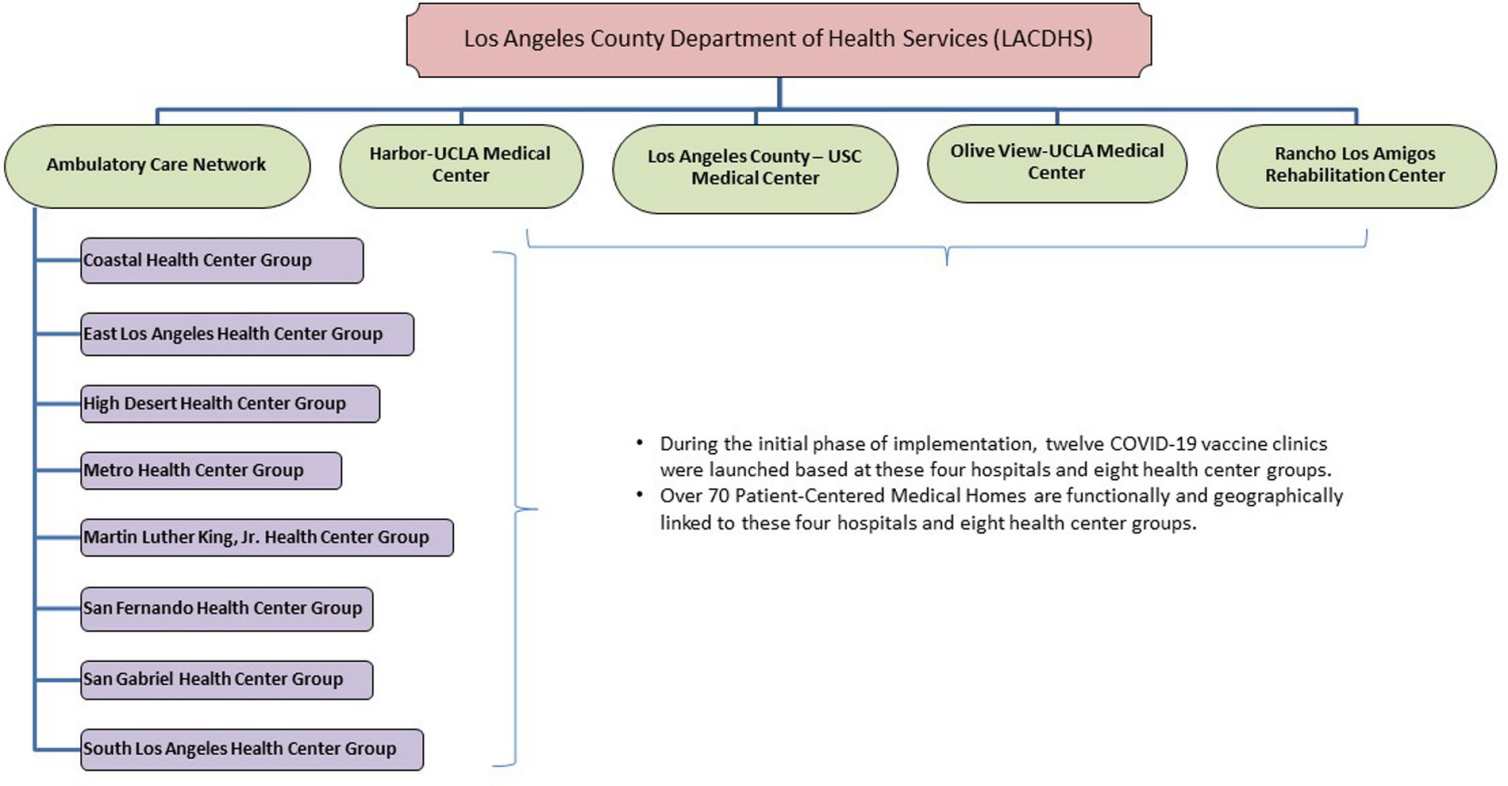
Los Angeles county department of health services (LACDHS) organization structure of COVID-19 vaccine clinics.

The Los Angeles County Department of Health Services (LACDHS) faced challenges in rapid vaccine implementation due to the size of the system, geographic breadth, language/racial/ethnic diversity and socioeconomic complexity of patients (low-income, publicly insured, and/or uninsured). LACDHS is the second largest public health system in the United States, serving over half a million unique patients annually across eight health center groups and four hospitals (Figure 1). Facilities span a geographic area greater than 4,000 square miles, including rural, urban, suburban, and exurban populations. The LACDHS empaneled patient population comprises approximately 60% Latinx and 12% Black/African American patients, compared to the overall LA County population with 46% Latinx and 8% Black/African-American people (10, 11). These populations were disproportionately affected during the pandemic as Latinx and Black people living in LA County had death rates nearly twice that of non-Hispanic white people (12–14). Persons in extremely poor or high-poverty census tracts had the highest COVID-19 case and death rates in LA County (14, 15).

In January 2021, LACDHS launched twelve COVID-19 vaccine clinics at each hospital and health center group, geographically spread across the county (Figure 1). The goal of the LACDHS COVID-19 vaccine implementation was to vaccinate as many patients as quickly as possible in the setting of limited access to vaccines, rapidly evolving eligibility guidelines, and staffing shortages related to the concurrent COVID-19 surge. We aimed to identify determinants impacting implementation of a systemwide COVID-19 vaccine intervention using the Consolidated Framework for Implementation Research (CFIR) in a safety net health system under circumstances where advance planning was limited. This evaluation could inform future population-level implementation efforts in safety net health systems, especially during public health emergencies.

## Methods

This qualitative study evaluated determinants of LACDHS COVID-19 vaccine clinic implementation during the initial period of phased vaccine availability from January 2021 to the end of April 2021. The LA County Department of Public Health (LACDPH) Institutional Review Board approved the study before the initiation of the research. We report our work using the Standards for Reporting Qualitative Research (16, 17).

### Setting and organization of LACDHS COVID-19 vaccine clinics

**Figure 2:**
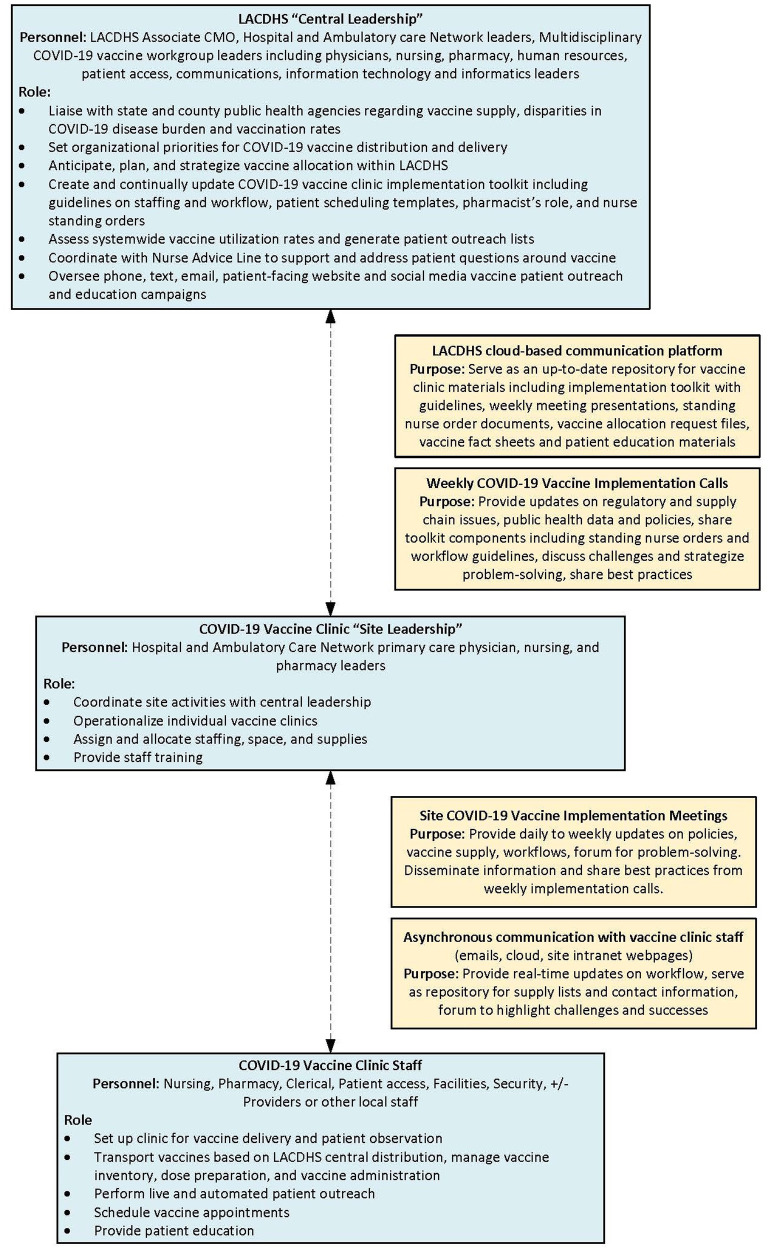
LACDHS COVID-19 vaccine clinic implementation leadership and communications structure. Blue-shaded boxes summarize leadership infrastructure and yellow-shaded boxes summarize communications infrastructure.

In January 2021, LACDHS leveraged its experience with and infrastructure from prior dedicated influenza clinic implementation to set up twelve COVID-19 vaccine clinics geographically spread across the county (Figure 1). LACDHS convened a multidisciplinary vaccine steering committee and primary care vaccine strategy workgroup to engage key stakeholders across disciplines and sponsor systemwide vaccination planning (Figure 2 “Central Leadership”). Each clinic site formed multidisciplinary leadership teams (Figure 2 **“**Site Leadership”) to oversee local COVID-19 vaccine clinic implementation.

Central leadership determined which sites within LACDHS would house its limited supply of ultra-low temperature freezers and met regularly to decide on system-level strategies impacting COVID-19 vaccine allocation and administration (Figure 2). Central leadership also made the critical decision to focus initial vaccination efforts on LACDHS-empaneled patients rather than the general public. Given that empaneled patients are disproportionately Latinx and Black, low-income, and undocumented people compared to the general LA County population, this was an intentional system-level decision to combat inequities in COVID-19 care.

COVID-19 vaccine clinics offered appointment-based and walk-in access. Some sites also offered drive-up services. To target eligible patients most effectively during the early phases of CDC vaccine eligibility, data-driven patient outreach lists were generated based on patient age and information on chronic conditions from the EMR. Utilizing outreach lists and scheduling scripts, clinic and call center staff called patients to schedule vaccine appointments, using bilingual staff when available. Additional strategies to reach vulnerable patients included automated phone calls, texts, and emails for high-volume population outreach. Staff also scheduled vaccine clinic appointments when patients presented to clinic sites for other reasons (i.e., primary care appointments, pharmacy medication pick-ups, and laboratory testing). Vaccine clinics were mostly staffed using existing staffing resources, with little additional hiring of contractors.

### Summary of LACDHS COVID-19 vaccine administration during implementation

From January 21, 2021, through April 30, 2021, LACDHS COVID-19 vaccine clinics administered 101,222 COVID-19 vaccinations. This number excludes doses administered in hospital inpatient wards, emergency departments, homeless sites, or correctional facilities not administrated by the COVID-19 vaccine clinics described herein. Of the 59,898 unique outpatients LACDHS vaccinated during this initial implementation period, 29.8% were aged 65 years or over, 69.7% were aged 18–64, and 0.5% were aged 16–17. 57.5% of vaccinated identified as female, 42.4% as male, and less than 0.1% as other or unknown. The race and ethnicity breakdown of those vaccinated during implementation was: Hispanic/Latinx 69.2%, Black/African American 8.1%, Asian 6.7%, White 3.6%, American Indian/Alaskan Native 0.1%, Native Hawaiian/Pacific Islander 0.1%, Multi-Race 0.3%, Other/Unknown 11.9%. The majority of COVID-19 vaccinations LACDHS administered (85%) went to individuals in the lowest two quartiles of the Healthy Places Index (accounting for social determinants of health including education, job opportunities, environmental factors) which surpassed overall LA County performance in this regard (18). Vaccine administration data and demographic information were gathered from the LACDHS electronic medical record and analyzed using Statistical Analysis Software.

### Interview guide

We utilized the CFIR to design an interview guide for COVID-19 vaccine clinic stakeholders. CFIR domains covered included inner setting, outer setting, individuals, process, and intervention characteristics (19, 20). The guide included open-ended primary questions and prompts to elicit thorough responses (see Supplementary Material A for the interview guide mapped to CFIR domains).

### Interview recruitment and methods

From August 2021 through November 2021, potential participants were recruited *via* purposive sampling. The research team asked the twelve vaccine clinic directors to identify a cross-section of personnel who played integral roles in the planning, managing, and/or daily operations of the local vaccine clinic from January-April 2021. These potential participants were either members of “Site Leadership” or front-line staff in COVID-19 vaccine clinics. Each potential participant was emailed up to three times. Interested participants were sent an electronic pre-interview demographic survey and attitude questions (see Supplementary Material B for pre-survey) and scheduled for an interview. Participants provided written informed consent. Interviews were recorded *via* HIPAA-compliant internet phone or video call and lasted 30–45 min. A professional transcription service transcribed interview recordings and de-identified proper names, clinic names, and locations.

### Rapid qualitative analysis

The research team applied a rapid analysis approach to analyzing all 25 stakeholder interviews (21–24). The team developed a master transcript summary template based on the original interview guide. This template was adapted upon the team trialing the template with six transcripts to improve standardization of data entry until consensus was reached. Researcher pairs then independently took notes, selected exemplar quotations from each de-identified interview transcript, compared notes, and edited a single high-level summary for each interview. Researchers met weekly to discuss findings, resolve discrepancies, and build consensus on transcript summaries. Each transcript summary was entered into an Excel matrix (24–27). Each row captured an individual interview and each column represented a topic area from the summary template (see Supplementary Material C for the transcript summary template). Then, the team identified and summarized major themes and representative quotes across interviews, which mapped to four CFIR domains. A synthesized summary of findings was presented to participants for comment and correction. An audit trail was kept throughout the analysis, including survey and interview guide drafts, scheduling logistics, raw data, field notes, rapid analysis summaries, weekly meeting minutes, and other process notes documenting key steps in methodological decision-making.

## Results

Of 40 potential participants, 32 responded to initial email requests, 26 completed the participant demographic survey, and 25 ultimately completed an interview (see Table 1 for Survey Participant demographics). The survey also included two questions related to the experience of participating in the implementation. When asked to rate the ease or difficulty of the COVID-19 vaccine implementation at their site on a Likert scale from 1 (easiest) to 5 (hardest), 31% (*n* = 8) of participants indicated implementation was easy or very easy and 38% (*n* = 10) participants indicated implementation was difficult or very difficult. Ninety-two percent of participants (*n* = 24) endorsed they would agree to be part of the COVID-19 vaccine clinic if asked again.

**Table 1:** Survey participant demographics*.

Role in LACDHS	% total surveyed (*n* = 26)	Count
Clinical provider/medical director	26.9%	7
Pharmacist	23.1%	6
Nursing staff	15.4%	4
Other (administrative, scheduling, health education, physical therapy)	34.6%	9
**Sex**		
Female	50.0%	13
Male	50.0%	13
**Age**		
20–30 years	3.8%	1
31–40 years	34.6%	9
41–50 years	30.8%	8
51–60 years	26.9%	7
61–70 years	3.8%	1
**Cultural background**		
Asian	42.3%	11
Latinx	26.9%	7
Caucasian	15.4%	4
Black/African American	11.5%	3
Native American/Pacific Islander	3.8%	1
**Years in current position**		
<1 year	3.8%	1
1–5 years	57.7%	15
6–10 years	11.5%	3
11–15 years	15.4%	4
16–20 years	3.8%	1
21–25 years	3.8%	1
>25 years	3.8%	1
**Vaccine clinic effort per week**		
1–10 h	26.9%	7
11–20 h	34.6%	9
21–30 h	7.7%	2
31–40 h	11.5%	3
>40 h	19.2%	5

* 26 staff completed the pre-interview demographics survey and 25 staff completed the interview.

Ten narrative themes emerged as determinants of the LACDHS COVID-19 vaccine implementation. These determinants are presented as they correspond to four CFIR domains: Innovation Characteristics, Outer Setting, Inner Setting, and Process. Themes and exemplar quotes are presented in Table 2.

**Table 2:** Themes and exemplar quotes from the Los Angeles County Department of Health Services COVID-19 vaccine implementation evaluation.

CFIR domain	Theme	Exemplar quotes from participants
Innovation characteristics	(1) LACDHS central leadership guidance coupled with local site flexibility supported problem-solving during rapid implementation.	• “DHS did a great job as far as giving us the guidelines and then what the facilities did was to take it and then make it their own. Like, we harmonized it.” (Clinical provider/medical director from Site 3) • “Everything was quite complicated. So, they took all those complex pieces and simplified it for us and made it into a workable system.” (Clinical provider/medical director from Site 1)
	(2) Multidisciplinary teams facilitated vaccine implementation and vaccine clinic activities.	• “ … it was a lot of collaboration with nursing, with providers, with pharmacy, and even down to the different departments … it was definitely a collaborative effort, and it was surprising how well it went in … I was impressed with DHS, actually, because it seems like everyone got together quickly. (Nursing staff from Site 11) • “DHS pharmacy staff was very supportive … if we needed to get more vaccine or get less vaccine, or swap vaccines, they were pretty open to it … sometimes it would require our pharmacy staff to travel from X to really, really far away, to XX probably one of the farthest away locations”. (Clinical provider/medical director from Site 12)
Outer setting	(3) Initial COVID-19 vaccine scarcity and rigid eligibility tiers led to ethical dilemmas.	• “So it was great to see them come but it was also sad to see people that were, like, 64 with the same conditions. ‘But yes, I’m sorry I can’t vaccinate you right now, but you’re in the next tier. Keep calling us, we will call you when we’re ready.’ But telling that to the community when they’re saying ‘I want it, I want it,’ and it’s a free vaccine, but we’re still following the CDC guidelines of holding it out.” (Clinical provider/medical director from Site 3) • “[We] felt very strongly that given this small, scarce supply, it was irresponsible for us as healthcare providers to let a dose go to waste. And so, my entire objective and purpose from the onset and being involved was to try to ensure that no dose was wasted.” (Other staff (administrative, scheduling, health education, physical therapy) from Site 4)
	(4) Initial COVID-19 vaccine scarcity and infrastructure limitations made coordination of vaccine delivery across LACDHS complex.	• “Initially it was little aliquots of vaccine coming through in certain ways for certain groups, so very restrictive criteria. Everything was quite complicated.” (Clinical provider/medical director from Site 1) • “Because you have this whole thing going on where you’re trying to predict and project your vaccine usage … So, I’m trying to project how many vaccines we’re going to do two weeks out, trying to make sure we’ve got the schedules and then trying see if we’re going to get full and actually use those. And we were routinely carrying over from one week to another because it just was impossible to hit it with the precision that they would have liked to” (Clinical provider/medical director from Site 10)
Inner setting	(5) Underestimated time and resources to overcome vaccine concerns and misinformation.	• “I discovered the amount of time required to engage with patients and incorporate the patient perspective, to understand where they’re coming from and to potentially get them to the point of being ready to get the vaccine. And so, to have these sensitive and challenging and energy-consuming conversations takes time. And I don't think that there was space made for that adequately.” (Other staff from Site 4) • “ … controversy with Johnson & Johnson came about and it was temporarily suspended and then it was restarted, but it was tough because people had already heard all this publicity and had concerns. But then you still had some people that were anxious to only have one shot. I mean we certainly tried to accommodate our patients as much as we could, but it’s a tough thing, overcoming vaccine hesitancy and not having the time to really work with all your outreach staff except at a very basic level on how to work with patients when they’re hesitant about being vaccinated.” (Clinical provider/medical director from Site 10)
	(6) Broader adoption and use of standing nurse order protocols enabled rapid capacity-building in COVID-19 vaccine clinics.	• “It was good … when they started rolling it out to more people to be trained for the [standing protocol] then we have more vaccinators and more staff that can help us with the clinic.” (Nursing staff from Site 2) • [Standing protocols] made it “a lot easier to give the vaccine to people. And it avoided us having to use the providers, which allowed them to do other things; so that was a good one.” (Clinical provider/medical director from Site 6)
	(7) A shared sense of purpose fostered a positive team culture.	• “ … That … concept of ‘It takes a village’ and our administration, everyone enacted an approach and commitment to getting our patients vaccinated.” (Clinical provider/medical director from Site 3) • “Since we’re there every day and working long periods of time, we all got to know each other very well, and it was a good mini family/team kind of ambience or vibe.” (Nursing staff from Site 3) • “Neat to be living through and involved with something that’s so big, and really momentous.” (Clinical provider/medical director from Site 5)
Process	(8) Active and passive communication structures enabled sites to adapt to evolving demands.	• “I had just an ongoing text message with the leads at the time just because you know, email was sometimes just not fast enough.” (Other staff from Site 7) • “At the end of the day we would also have a post-clinic huddle where we would talk about what went well today or what could have been improved or things that happened on that day, like, that clinic, and then ways we can improve them for tomorrow.” (Nursing staff from Site 1)
	(9) Developed patient-centered engagement strategies for COVID-19 vaccine clinic scheduling and vaccine administration.	• “So the patients who needed to be in their cars, so they were handicapped, they weren’t dressed for the weather, they had a child—and this are all that has happened—or they were helping someone who was not ambulatory or they themselves weren’t ambulatory. We set up, like, reserved parking spaces as close to the vaccine station as possible and they would just tell the registration staff when they drove in that they were not able to walk up.” (Other staff from Site 7) • “We were trying to spread the word that, hey, our site is offering COVID vaccines and … that population where there’re a lot of African Americans. And I know based on the history, there’s a lot of resistance or hesitancy. So there was a lot of outreach done and I think that’s why a lot of the nurses, they continued to call the day before and the day of their vaccination appointment just to confirm that they’re going to keep it and also to answer any questions.” (Pharmacist from Site 9)
	(10) Sites encountered a variety of process challenges implementing COVID-19 vaccine clinics.	• “So the way that we had set up this clinic, it was actually in the older parking garage … something so simple as that. That’s where we were actually stationed. But then if there was rain, we would feel the rain. So then, we would have to quickly shift somewhere else within the clinic. I mean, if we’re vaccinating … 200 patients within a few hours, so of course it could be a little tight for spacing.” (Nursing staff from Site 11) • “ … then you have this challenge of people working one list and then you get another list and a lot of it’s still duplicative and cumulative … Then you have the other challenge of internally somebody having to take that list and put it on some sort of shared drive or something because you might have multiple people working the same list. And you get some feedback that patients are getting tired of getting calls about this … so trying to document that this person doesn’t want any more calls … ” (Clinical provider/medical director from Site 10)

### Innovation characteristics—implementation of COVID-19 vaccine clinics

Theme 1. LACDHS central leadership guidance and local site flexibility supported problem-solving during rapid implementation.

The LACDHS leadership communication infrastructure included structured weekly and *ad hoc* meetings between central and site leadership (Figure 2). This served as a platform for the bidirectional exchange of ideas between central and site leadership and across sites. These meetings provided a forum to clarify rapidly evolving information, coordinate and align around promoting health equity, and share best practices and lessons learned which site leaders could bring back to vaccine clinics to adapt local workflows quickly. Sites had leeway to adapt workflows based on local needs and resources while aligning with central guidelines. Participants viewed central leadership as informative and transparent, communicating regularly to inform clinics of the latest federal, state, and county policies.

Standardization worked effectively to an extent, but it was ultimately up to the clinics to adapt the implementation to best meet the needs of their local site and teams, particularly related to staffing and space availability. Participants described variations across vaccine clinics regarding infrastructure, demonstrating local flexibility in implementing central guidance. Sites strategized staffing solutions in the context of a concurrent winter COVID surge with nursing shortages due to frequent staff sick calls and redeployment of outpatient nursing to inpatient settings. As a result, some sites used staff from pandemic-closed clinics (e.g., dentists), some paid overtime for staff to work additional hours, and others used registries, volunteers, or students to staff vaccine clinics. Some sites pulled staffing from primary care or urgent care clinics, leaving those clinics short-staffed, sometimes leading to staff resentment. Each site had different vaccine clinic floor plans with varying accessibility to host the vaccine clinics. Some sites held the vaccine clinics indoors in temporarily closed clinics or repurposed spaces, including lobbies and auditoriums, and others held clinics outdoors on sidewalks, patios, and in covered garages.

Theme 2. Multidisciplinary teams facilitated vaccine implementation and vaccine clinic activities.

LACDHS assembled a multidisciplinary leadership team at the central level which included physician, nursing, pharmacy, and patient access leadership (Figure 2). This team designed a vaccine implementation toolkit to provide integrated guidance for the vaccine clinics. For example, physician leadership digested and communicated clinical and public health updates, nursing leadership addressed workflow and informatics needs related to vaccine administration, and patient access leads designed scripts and workflows for outreach and patient registration. Due to the complexity of inventory and allocation, storage, handling, and preparation of the vaccines, pharmacy leadership coordinated the distribution of large direct vaccine shipments across the system and monitored utilization across vaccine clinics. Pharmacy leads managed re-distribution of vaccine between sites to accommodate daily patient volume and minimize waste associated with short expiration dates.

Based on central leadership toolkit guidelines, site leadership assembled local multidisciplinary teams to problem solve and optimize workflows across staff types, and adapt workflows in real time. The strategic choice to designate a lead pharmacist role in the COVID-19 vaccine clinics was identified as an essential enabler of vaccine clinic efficiency as site pharmacists had knowledge of LACDHS vaccine resources and could mix, draw, administer, and counsel on the vaccine. Central leadership toolkit materials were designed to allow for workflow and role flexibility. For example, pharmacists could administer vaccine if there were nursing shortages, and nursing could register new patients when there was a clerical shortage.

### Outer setting—macro-level factors that originate outside the LACDHS system

Theme 3. Initial COVID-19 vaccine scarcity and rigid eligibility tiers led to ethical dilemmas.

During the early weeks of vaccine scarcity and strict adherence to state and federal eligibility tiers, avoiding vaccine wastage was one of the participants' most significant concerns and even a source of anxiety. This felt most weighty at the end of a clinic session when the time came to draw the vaccine from the last multi-dose vial for the day, and there were more doses than patients remaining. Some participants expressed this as an ethical challenge: avoiding administering vaccines to patients outside the eligibility tiers meant doses might be wasted. Participants noted it was challenging to stay within eligibility tiers as tiers rapidly shifted. For example, at one point, there was discordance between the CDC and LACDPH guidance on the definition of chronic conditions and how to vet eligibility by occupation instead of age. Participants also experienced moral conflict when withholding vaccine from high-risk patients close to meeting eligibility criteria but did not fall into current eligibility tiers. Participants noted this was a tense time—balancing a reluctance to turn patients away with the risk of vaccine wastage resulted in extraordinary efforts to find patients to use the last remaining doses which could not be stored. One participant described this undertaking:

*“We felt very strongly that given this small, scarce supply, it was irresponsible for us as healthcare providers to let a dose go to waste. And so, my entire objective and purpose from the onset and being involved was to try to ensure that no dose was wasted.”—*Other staff member (administrative, scheduling, health education, physical therapy) from Site 4

To administer all remaining doses, participants performed last-minute outreach including overhead announcements, finding vulnerable staff such as environmental services and dietary workers to vaccinate, or going to Urgent Care and the emergency department to find patients before the vaccine had to be wasted.

Theme 4. Initial COVID-19 vaccine scarcity and infrastructure limitations made coordination of vaccine delivery across LACDHS complex.

Initial scarce COVID-19 vaccine supply necessitated complex coordination of vaccine distribution across our large health system. LACDHS received vaccine shipments weekly only at select sites with ultra-low temperature freezers. Vials then had to be re-distributed to sites without ultra-low freezers. Limited and variable weekly vaccine allocations restricted how far in advance patients could be scheduled. This resulted in complicated staffing and outreach planning, and sometimes led to site pharmacists driving long distances across the county to pick up doses from another LACDHS site. Participants also commented on the challenges of dealing with unpredictable and variable vaccine availability and the differences across multiple vaccine brands, including dosing intervals, expiration dates, and community preferences.

### Inner setting—pertaining to the infrastructure, resources, and culture of the LACDHS system

Theme 5. Underestimated time and resources required to overcome vaccine concerns and misinformation.

Participants observed that additional time and resources were needed to overcome vaccine hesitancy and misinformation at all points of patient contact. This included encounters with the primary care provider, the nurse advice line, at the time of vaccine scheduling, while waiting in line at the vaccine clinic and at the time of vaccine administration. Local sites performed most of the patient-level vaccine outreach and scheduling mostly using non-clinical call center staff. However, non-clinical staff felt unprepared to answer vaccine questions from patients. Some sites reassigned clinical staff to make individualized calls to vaccine-hesitant patients or answer questions on-site at the vaccine clinics. Participants desired more community education and outreach and perceived a lack of consistent scripting for staff, especially in addressing complex vaccine conversations during a clinic visit with several competing priorities. Patients presenting to the vaccine clinic intending to get vaccinated still had questions about allergies, interactions, what to expect after the vaccine, and other concerns. Some sites created their own patient education and FAQ materials. It was viewed as a barrier that LACDHS central leadership did not provide more support in this area. A few staff expressed concern for their own safety from exposure to patients with COVID-19, and compared the COVID-19 pandemic to the HIV epidemic

Theme 6. Broader adoption and use of standing nurse order protocols enabled rapid capacity-building in COVID-19 vaccine clinics.

Participants recognized that the urgency and breadth of COVID-19 vaccine implementation warranted a transformation of existing workflows to improve efficiency for widespread vaccination. A meaningful change was delegating provider vaccine ordering authority to nursing staff for quicker vaccine ordering. LACDHS had prior experience with standing nurse orders, however an important change was made to the nurse training process for the COVID-19 vaccine implementation. Training for the standing nurse orders shifted from periodic in-person training to on-demand recorded virtual training for nurse vaccinators, which allowed hundreds of vaccinators to be trained in a short amount of time. Additionally, electronic post-training proficiency testing allowed for real-time calculation of results, which were posted to the staff portal where an up-to-date master roster of staff ready to vaccinate was maintained. Participants thought completing the online training before arriving to work at the vaccine clinic facilitated orientation and same-day onboarding while staffing was in flux.

Theme 7. A shared sense of purpose fostered a positive team culture

Participants noted a fellowship with their vaccine clinic co-workers when asked about site-level engagement. Staff had a strong sense of purpose and a feeling of responsibility to match the moment and be a part of history fighting the pandemic. Participants agreed a robust process for communication and collaboration amongst the local site team was a key factor in success. Participants desired to reach as many patients as possible with an “all hands-on deck” approach and willingness to do whatever it took to “make it work.” One participant described the team approach:

*“That … concept of ‘It takes a village’ and our administration, everyone enacted an approach and commitment to getting our patients vaccinated.”—*Clinical provider/medical director from Site 3

Multiple participants expressed feeling proud that they were making a difference. Openness to feedback and continuous improvement fostered a culture of multidisciplinary teamwork and collaboration, which stemmed from shared investment in the work. Sites were keenly aware of the safety net patient population, which led to many discussions at the local level about historical and contemporary marginalization and vaccine hesitancy as barriers to COVID-19 health equity. This strong sense of purpose facilitated buy-in for COVID-19 vaccine clinic implementation. Additionally, participants praised site leaders who showed gratitude and appreciation for vaccine clinic staff. Many leaders were present on the front lines to quickly address staffing and supply issues, effectively promoting a teamwork culture.

### Process—means by which LACDHS COVID-19 vaccine clinics were implemented

Theme 8. Active and passive communication structures enabled sites to adapt to evolving demands.

Participants discussed the site-specific rapid decision-making related to implementation of brand-new vaccine clinic workflows and expressed the feeling that “we were building the plane as we flew it*.*” Site leaders realized they had to develop site-specific tools and infrastructure to support real-time communication between local team members. Debriefing with frontline staff promoted staff engagement in continuous improvement and enabled sites to walk back from stalled innovations. Sites with effective communication used various tools (e.g., emails and remote meeting platforms) and built redundancy in their communication structure (e.g., daily clinic huddles, weekly meetings, and workstations in proximity to leaders). Sites without timely, broad, and multidisciplinary communication structures felt challenged. Participants cited *ad hoc* meetings, frequent updates relaying messages from central leadership, and openness to feedback from frontline staff as effective communication methods used by site leaders.

Theme 9. Developed patient-centered engagement strategies for COVID-19 vaccine clinic scheduling and vaccine administration.

Participants enthusiastically described the novel ways their sites engaged patients to get vaccinated.

LACDHS central leadership created low-literacy vaccine Frequently Asked Questions documents in English and Spanish for use in the vaccine clinics. Site leaders were intentional about staffing vaccine clinics with diverse and multilingual staff and interpreters, along with providing appropriate educational materials when available. Sites used data-driven patient outreach lists provided by central leadership to schedule patients. Motivational interviewing, clinic staff sharing their vaccine stories, and face-to-face patient communication were important tools that helped engage patients. Sites prioritized direct patient education and communication; providers and clinic staff engaged patients while waiting in line and during and after vaccine administration to answer questions.

Sites provided broad access to vaccine appointments by offering evening and weekend clinics, accepting walk-ins, and performing patient-centered scheduling to combine a vaccination visit with another clinic visit. Online self-scheduling was also available systemwide for first doses. Participants expressed a desire for expanded self-scheduling for subsequent vaccine doses. Efforts to recruit patients for vaccine scheduling extended beyond phone outreach to every touch patients had with the clinics, e.g., picking up medications at the pharmacy or getting labs done. Participants described strategies to meet limited-mobility patient needs by providing wheelchairs and walkers on-site, vaccinating at curbside, and coordinating home vaccination referrals. One participant described these efforts:

*“So the patients who needed to be in their cars, so they were handicapped, they weren’t dressed for the weather, they had a child—and this are all that has happened—or they were helping someone who was not ambulatory or they themselves weren’t ambulatory. We set up, like, reserved parking spaces as close to the vaccine station as possible and they would just tell the registration staff when they drove in that they were not able to walk up.”—*Other staff member from Site 7

Additionally, patient safety, comfort, and experience were of paramount importance. Participants reported designating places for patients to lie down, socially distanced observation areas, and providing free personal protective equipment and outdoor heating.

Theme 10. Sites encountered a variety of process challenges implementing COVID-19 vaccine clinics.

Central leadership designed social media and broadcast message campaigns in English and Spanish to encourage vaccination and created patient outreach lists stratified by language for sites to schedule eligible patients. While most sites agreed with this outreach approach, one site refrained from performing language-concordant outreach for fear of prioritizing that ethnic group over English-speaking patients. The live outreach calls required extensive effort, yielded mixed results, and sometimes seemed to be wasted effort. Non-clinical scheduling staff worked outreach lists that were thousands of patients long, making multiple attempts and leaving voicemail messages if patients did not initially answer. Site staff also leveraged previously infrequently used robocall technology to perform automated outreach. Staff accommodated variable incoming call volume by adjusting staffing shifts, modifying the interactive voice response (phone tree branching structure), and continuously monitoring calls. In addition to this outreach, sites fielded a high volume of incoming calls from patients requesting to schedule vaccine appointments, many of whom were not yet eligible per county eligibility tiers.

LACHDS COVID-19 vaccine clinics were based in primary care settings. The hospitals and larger health centers also deliver specialty care and varied in how much their vaccine clinic collaborated with specialty care. These sites varied in their workflows of how patients in specialty care were directed to vaccine clinics, and how limited staffing was distributed between primary care, vaccine, and specialty care clinics. During the early phases of implementation in the setting of vaccine scarcity, central leadership focused initial vaccination efforts on LACDHS-empaneled primary care patients rather than the public to promote vaccine equity. This led to some confusion and tension at sites when non-empaneled patients receiving specialty care at LACDHS sites could not be vaccinated even when meeting vaccine eligibility criteria.

Participants were forthcoming that not all site-level operational decisions were adaptive. Examples of workflow decisions that were not sustained or served as barriers to efficient vaccination included: not hiring temporary nursing staff which resulted in staffing shortages, mixing vaccines in pharmacy hoods rather than at the vaccine clinic which required additional staff runners to transport vaccines, limiting Janssen vaccine administration for women over age 50 due to concern for thrombus despite no such FDA guideline, and not opening vaccine clinic on county holidays despite available staffing. Some participants noted that central leadership could have helped anticipate some logistical needs of the sites, such as coordinating bulk printing of vaccine clinic signage and purchasing of cold cubes for vaccine storage and tents for outdoor vaccine administration.

## Discussion

Participants’ experience of the LACDHS COVID-19 vaccine implementation converged on ten themes related to four CFIR domains: Innovation Characteristics, Inner Setting, Outer Setting, and Process. These themes illustrate how our large safety net health system rapidly mobilized to launch broad-scale COVID-19 vaccination during a public health emergency. Limited resources necessitated LACDHS leadership and staff to be resourceful by leveraging bi-directional communication, quickly adapting to local site and patient needs, and promoting teamwork, all while aligning work to evolving COVID-19 vaccine guidelines.

Our study adds to the literature by providing a qualitative assessment of a large-scale implementation in a safety net health system where coordination across many sites was a core feature. LACDHS had implemented systemwide programs in the past (28, 29); however, no previous intervention was as far-reaching or had to be rolled out as quickly under such a systemwide strain on resources. To date, there have been few publications detailing COVID-19 vaccine implementation in safety net systems. DiVirgilio et al. emphasized community education and targeting by zip code to focus on communities disproportionately affected by COVID-19 morbidity and mortality in their Chicago study (8). San Francisco's safety net health network highlighted drop-in hours as the most effective way to lower barriers to COVID-19 vaccine access (9). Both studies operated on a smaller scale (approximately 5,000 patients in the San Francisco study and 11,000 patients in the Chicago study), with less geographic breadth.

A key facilitator of implementation seen in some safety net health system studies is an emphasis on advance planning for implementation (5, 6). However, health systems did not have the luxury of advance planning with COVID-19 vaccine implementation during the pandemic. The LACDHS case of COVID-19 vaccine clinic implementation under time pressure suggests that real-time frontline staff input into implementation design and balancing system standardization and local site adaptations are important facilitators in scenarios where advance planning is not possible. The rapid stand-up of LACDHS COVID-19 vaccine clinics demonstrates how a traditionally rigid system can be agile and adaptive to meet the moment.

A recent review of qualitative implementation studies in safety net settings found that understaffing and high staff turnover rates were the most common reason for the lack of acceptability of interventions (6). LACDHS, like other safety net systems, experienced high staff turnover and understaffing rates during the pandemic due to the inpatient COVID-19 surge and COVID-19-related sick callouts. Our study identified the use of multidisciplinary teams, bidirectional communication across leadership and sites, and the broad use of standing nurse orders as facilitators which helped overcome the barriers of staff turnover and understaffing. Similar to Crable's findings (3) where stakeholders' personal commitment to reduce health inequities was a facilitator of implementation, a takeaway of this evaluation is that a positive work culture and a clear shared goal helped mitigate a pressured work environment with high demands. Our themes of the importance of bidirectional and frequent communication and addressing patients' biopsychosocial complexities also aligned with previous studies of implementation in safety net settings (3).

The LACDHS vaccine clinic implementation deepened participants' and the research team's appreciation for the social complexity of the LA county safety net population. Frontline staff served as the best advocates for identifying and addressing social needs to lower barriers to patient vaccine access (30); however, beyond providing operational accommodations such as expanded vaccine clinic hours, bilingual staff, and assistance for those with limited mobility, sites had varying interpretations of how to promote health equity in vaccine clinics. Most sites and staff naturally focused on addressing the social needs of individual patients, rather than on the root causes of health inequities. Participants experienced moral discomfort when asked to focus on empanelment status and vaccine eligibility criteria, rather than vaccinating all-comers. These scenarios illustrate how additional training promoting a deeper understanding of health equity vs. equality is needed. To build on a commitment to inclusive care, LACDHS should offer staff training to further develop structural competency in health equity, an important step in the journey to advance health equity (31–33).

The LACDHS COVID-19 vaccine implementation experience highlighted the need to develop a comprehensive patient education strategy at the system level, encompassing outreach communications as well as education at the point of care. Safety net patient populations comprise diverse groups with different sociocultural, education, and outreach needs to combat vaccine misinformation and promote vaccine uptake (34–37). Participants expressed that central leadership did not provide enough support in patient education. LACDHS central leadership included low-literacy vaccine FAQ documents and scheduling scripts in its implementation toolkit, however, additional support and a robust infrastructure for patient education was needed. Some sites opted to create their own scheduling scripts, FAQs, and signage, reallocated clinical staff to address questions, and attempted to use language-concordant and culturally concordant staff to promote optimal health communication. Additional patient education materials in the vaccine clinics and resources to address vaccine hesitancy might have increased vaccine acceptance and vaccination rates. Allocating funding for and investing resources in building patient education, engagement, and communications infrastructure as a core service for safety net systems would be an important step to laying the foundation for future successful safety net implementations.

Study limitations include potential participant recall bias. Interviews were conducted from September to November 2021, months after the defined early vaccine implementation period from January to April 2021. Interviews were intentionally conducted mainly with vaccine clinic team members rather than central leadership, leading to a perspective focused more on site and frontline experiences. Finally, this study lacked patient perspectives on the LACDHS vaccine clinic implementation.

This comprehensive qualitative analysis of the LACDHS rapid implementation of COVID-19 vaccine clinics yielded important lessons for safety net health systems caring for populations experiencing disproportionate disease burden due to societal inequities. This analysis fostered a deepened understanding of facilitators which can help overcome understaffing and a lack of advance planning. Key facilitators included using robust communication between all levels of the organization and balancing workflow standardization with local site flexibility. Additional lessons included the importance of building system capacity for health equity work and regarding patient engagement and communications infrastructure as a core necessity for safety net health systems. Applying these lessons in future implementations can benefit staff, patients, and safety net communities.

## Data Availability

The original contributions presented in the study are included in the article/**Supplementary Material**, further inquiries can be directed to the corresponding author.
