# Corrigendum: Evaluation of COVID-19 vaccine implementation in a large safety net health system

**DOI:** 10.3389/frhs.2025.1627499

**Published:** 2025-05-29

**Authors:** Jennifer C. Chen, Griselda Gutierrez, Rachel Kamran, Jill Terry, Armenui Telliyan, Camilo Zaks, Savanna L. Carson, Arleen Brown, Karen Kim

**Affiliations:** ^1^ Ambulatory Care Network, Los Angeles County Department of Health Services, Los Angeles, CA, United States; ^2^ Department of Emergency Medicine, David Geffen School of Medicine at UCLA, Los Angeles, CA, United States; ^3^ Harbor-UCLA Medical Center, Los Angeles County Department of Health Services, Los Angeles, CA, United States; ^4^ Department of Obstetrics and Gynecology, David Geffen School of Medicine at UCLA, Los Angeles, CA, United States; ^5^ Fielding School of Public Health Graduate Program, University of California, Los Angeles, CA, United States; ^6^ Pharmacy Affairs, Los Angeles County Department of Health Services, Los Angeles, CA, United States; ^7^ Adjunct Faculty, USC School of Pharmacy, Los Angeles, CA, United States; ^8^ Adjunct Nursing Faculty, Glendale Community College, Glendale, CA, United States; ^9^ Adjunct Nursing Faculty, West Coast University, Los Angeles, CA, United States; ^10^ Division of Street Medicine, Keck School of Medicine of USC, Los Angeles, CA, United States; ^11^ Division of General Internal Medicine & Health Services Research, David Geffen School of Medicine at UCLA, Los Angeles, CA, United States; ^12^ Population Health Management, Los Angeles County Department of Health Services, Los Angeles, CA, United States; ^13^ Department of Medicine, David Geffen School of Medicine at UCLA, Los Angeles, CA, United States

**Keywords:** COVID-19, vaccine, vaccine distribution, implementation, leadership, communication, integrated delivery of health care, equity

A Corrigendum on Evaluation of COVID-19 vaccine implementation in a large safety net health system By Chen JC, Gutierrez G, Kamran R, Terry J, Telliyan A, Zaks C, Carson SL, Brown A, Kim K (2023). Front Health Serv. 3:1152523. doi: 10.3389/frhs.2023.1152523

In the published article, there was an error in the Funding statement. Instead of “This paper was supported by NIH/NHLBI CEAL grant #21-312-0217571-661106l and NIH/NCATS grant #uL1TR0018812, it should have read “This research was, in part, funded by the National Institutes of Health (NIH) Agreement OT2HL158287”. The correct Funding statement appears below.

**FUNDING**

This research was, in part, funded by the National Institutes of Health (NIH) Agreement OT2HL158287. The views and conclusions contained in this document are those of the authors and should not be interpreted as representing the official policies, either expressed or implied, of the NIH.

The authors apologize for this error and state that this does not change the scientific conclusions of the article in any way. The original article has been updated.

