# Supplementary material for: Evaluation of COVID-19 vaccine implementation in a large safety net health system
Source: Front Health Serv. 2023 Jun 5;3:1152523. doi: 10.3389/frhs.2023.1152523 (PMC10277563; doi:10.3389/frhs.2023.1152523)
Supplement: Supplementary file 1 [file Table1.docx]

| **Construct** | **Questions** | **Probes** |
| --- | --- | --- |
| Evidence Strength & Quality/  Knowledge and Beliefs about the Intervention/  Reflecting and Evaluating | Today we want to learn about what went well and what could have been improved with the DHS patient COVID-19 vaccine express clinic implementation. We’re focusing particularly on the January to May 2021 period, including from when we started building the DHS patient COVID-19 vaccine Express clinics, expanding and scaling up vaccine clinic access, adding eligible tiers/groups, and the different brands of vaccines along the way.  With that, can you please share your thoughts on your site’s patient COVID-19 vaccine express clinic implementation? | How do you think the implementation went?  Over that period of time, how did you know if things were going well or not? For instance, what information did you have to assess how the COVID vaccine clinic implementation was going? Data, guidelines, conversations with others, other feedback or information?  Did you feel this rollout was important to your community and the communities you serve? |
| Innovation Source (DHS Central Coordination) | The rapid vaccine rollout timelines and government allocation process required some DHS central coordination for the launch of the COVID-19 Vaccine Express clinics.  In what ways did DHS central coordination of the Express clinics meet or not meet your site’s needs? | Can you give examples of DHS central coordination that you found particularly helpful? (weekly phone calls, guidelines, previous implementation projects, etc.)  What are other ways that DHS vaccine leadership team might have supported your site? |
| Adaptability | As your site was working on the vaccine rollout, what flexibility did your site have to make changes along the way? What flexibility to make changes did you have as an individual? | Can you give some examples of what about the program was flexible to help meet rollout needs?  Were there adaptations you wanted to make but were unable to?  What was the process for deciding on whether changes were needed to adapt for your site?  Would more or less flexibility have been helpful and why? |

| Patient Needs & Resources | How were the needs and preferences of DHS patients considered during implementation? | How equitable was the rollout to the communities you serve? How could equity have been improved?  What specific barriers to vaccination did DHS patients face? (eg.Location? Language? Hours/days? Websites? Information? Other?) |
| --- | --- | --- |

| Relative Priority | At your site, how did you prioritize implementation of the COVID vaccine express clinic? | Did the COVID vaccine clinic implementation conflict with other high-priority activities at your site? If so, how?  How was this prioritization perceived differently across different service lines or departments at your site? |
| --- | --- | --- |
| Available Resources/access to knowledge & information | Did you have sufficient resources to implement and administer the COVID vaccine express clinic? What resources did you have access to and what did you find you needed more of? | Prompts: Staffing, PPE, computers, information, etc.?  Who did you ask if you had questions? |
| Leadership Engagement | Tell us about leaders at your site and at the DHS level for the COVID-19 express clinic. How would you describe the involvement of leaders and managers? | What kind of support did they provide? Specific examples.  Did they provide feedback? What kind of feedback?  Were they too involved or not involved enough? Why? |
| Engaging | How did your site engage staff (both front line and local leaders) in the implementation of your COVID vaccine express clinic? What worked and what could have been improved about communication with staff about the COVID vaccine express clinic at your site? | Prompts: meetings, education, training, feedback and reflection, other activities |
| Networks & Communication/  Key Stakeholders | What worked and what could have been improved in terms of communication with patients about the COVID vaccine express clinic at your site? | Prompts: written communication, outreach |
|  | Is there anything else you want to add about how the vaccine express clinics went?  Any questions we should have asked and didn’t? |  |
