# Supplementary material for: Evaluation of COVID-19 vaccine implementation in a large safety net health system
Source: Front Health Serv. 2023 Jun 5;3:1152523. doi: 10.3389/frhs.2023.1152523 (PMC10277563; doi:10.3389/frhs.2023.1152523)
Supplement: Supplementary file 2 [file Table2.docx]

Pre-Interview Questions

Dear Interviewee,

Thank you for your participation in this project.  We value your contribution to help understand how DHS performed in its implementation of the COVID-19 Patient Vaccination Campaign.  The study objective is to learn and describe what went well, and importantly, what could be improved, so we can use this as a model for future system-wide endeavors.

**Prior to your scheduled interview**, please complete this confidential questionnaire.

**Current role:**

**□**Administrator **□**Doctor or Nurse Practitioner **□**Health Educator **□**Nurse       **□**Pharmacist  **□**Not Listed:_______________________________________________

**Gender:**

**□**Female **□**Male **□**Trans Woman  **□**Trans Man

**□**Gender Non-Conforming  **□**Not Listed:___________________________________

**Race/Ethnicity (check as many as apply)**:

**□** Asian   □ Black / African American □ Caucasian / White

□ Hispanic / Latino/a/x □ Native American/Alaskan □ Native Hawaiian / Pacific

**Age:**

**□ <**20 years **□**20 –30 years **□**31 – 40 years **□**41 – 50 years

**□**51 – 60 years     **□**61 – 70 years **□**71 – 80 years   **□**>80 years

**How long have you worked in your current position?**

**□**< 1 year    **□**1-5 years **□**6-10 years **□**11-15 years

**□**16-20 years **□**21 – 25 years **□**>25 years

**From January 2021 – May 2021, how much time did you spend on PATIENT COVID-19 Vaccination efforts? (average hours per week including planning, managing, and/or staffing)**

**□**< 5 hours    **□**6-10 hours **□**11-15 hours **□**16-20 hours   **□**21-25 hours **□**26 – 30 hours **□**31 – 35 hours **□**36 – 40 hours  **□**>40 hours

***Please circle the response that best describes your opinion on the following statements****.*

| **Question** | **1** | **2** | **3** | **4** | **5** |
| --- | --- | --- | --- | --- | --- |
| How would your rate the ease or difficulty of the patient COVID-19 vaccine implementation at your site? | Very  Easy | Easy | Not sure | Difficult | Very  Difficult |
| If asked again, I would agree to be a part of the COVID Vaccine Express Clinic ? | Definitely  Yes | Yes | Not sure | No | Definitely   No |

Who led the COVID-19 Vaccine Implementation at your site? (Please include name and position)

 ______________________________________________________________________________________________________________________________________________________________________________

You have completed the pre-interview questions.  Thank you for your responses.
